# Association of High Serum Adiponectin with the Risk of Malnutrition and Worse Outcome in Head Trauma Patients; a Cohort study

**Published:** 2019-08-24

**Authors:** Mohammed Ibrahim Mohialdeen Gubari, Mohammad Javad Hosseinzadeh-Attar, Mostafa Hosseini, Fadhil Ahmed Mohialdeen, Abdolreza Norouzy

**Affiliations:** 1 Department of Clinical Nutrition, School of Nutritional Sciences and Dietetic, Tehran University of Medical Sciences, Tehran, Iran.; 2 Centre of Research Excellence in Translating, Nutritional Science to Good Health, The University of Adelaide, Adelaide, Australia.; 3 Department of Epidemiology and Biostatistics, School of Public Health, Tehran University of Medical Sciences, Tehran, Iran.; 4 Community Health Department, Technical College of health, Sulaimani Polytechnic University, Sulaimani, Iraq.

**Keywords:** Adiponectin, craniocerebral trauma, body composition, APACHE, organ dysfunction scores

## Abstract

**Introduction:**

A number of studies have shown the association between serum adiponectin level and the nutritional status. This study aimed to evaluate the relation between serum adiponectin and changes in nutritional status of head trauma patients.

**Methods:**

The current prospective cohort study was carried out on head trauma patients who were hospitalized in ICU of a General Teaching Hospital, Sulaimani, Iraq. Patients were divided into two groups based on their serum adiponectin level during the first 24 hours of admission (<15mg/L and ≥15 mg/L) and malnutrition and nutritional indices were compared between groups 1, 6 and 13 days after admission.

**Results:**

Sixty-four patients with the mean age of 35.97 ± 11.5 years were studied (59.4% male; 57% traffic accidents). The nutritional status of head trauma patients with serum adiponectin ≥15 mg/L significantly deteriorated from day 1 to 13 based on different nutritional status indices. BMI (p = 0.08), LBM (p = 0.002), APM (p = 0.009), and MUAC (p = 0.008) had a significant decreasing trend from day 1 to day 13 in patients with serum adiponectin level ≥ 15 mg/L. In addition, the number of high risk patients for developing malnutrition based on NUTRIC score (p < 0.001) and the number of severely malnourished cases based on SGA score (p < 0.001) significantly increased from day 1 to 13 in this group. The severity of disease based on APACHE (p < 0.001) and SOFA (p < 0.001) scores increased in the mentioned cases during the follow up period.

**Conclusion:**

Serum adiponectin level ≥ 15 is associated with significant deterioration in nutritional status, increase in the risk of malnutrition, and worsening of the clinical outcome in patients with moderate to severe head trauma in ICU.

## Introduction

Adiponectin is one of the bioactive proteins produced by white adipose tissue with a role in the energy homeostasis, lipid and glucose metabolism, and an anti-inflammatory activity (1). A high level of serum adiponectin is linked to cardiovascular diseases, metabolic disease, rheumatoid arthritis, and inflammatory bowel disease, whereas, a low level of serum adiponectin is linked to brain and myocardial infarctions (2, 3). A high concentration of adiponectin has been linked with increased rate of mortality in both cardiovascular diseases and stroke (4). Increase in adiponectin has been observed not only in chronic diseases but also in patients with severe traumatic brain injury (5). The association between serum adiponectin and the nutritional status of haemodialysis patients is stated (6). The level of adiponectin also plays a role in pathogenesis of cachexia in heart failure (7). High level of adiponectin might lead to the loss of body weight in an attempt to normalize the metabolism of fatty acid. In animal model, administration of adiponectin has been linked with increase in energy expenditure, causing weight loss (8).

There is limited data on the association between circulatory adiponectin levels and nutritional status in head trauma patients. Therefore, this study was conducted to investigate the relationship between serum adiponectin and changes in nutritional status of head trauma patients. 

## Methods

***Study design and setting***

The current prospective cohort study was carried out on head trauma patients who were hospitalized in ICU of a General Teaching Hospital of Sulaimani located in the Kurdistan Region of Iraq, from 20 November, 2017 to 07 August, 2018. Patients were divided into two groups based on their serum adiponectin level in the first 24 hours of admission (<15mg/L and ≥15 mg/L) and malnutrition and nutritional indices were compared between groups 1, 6 and 13 days after admission to ICU. 

This study was carried out according to the standard clinical ethics guideline, and its ethical approval was obtained from the Ethics Committee of Tehran University of Medical science (TUMS) with the code IR.TUMS.VCR.REC1396.2676. Moreover, after the target patients and their families were provided with explanations about the study’s method and objectives, confidentiality of the data, and their freedom to quit the study, their written consent was obtained.

***Participants***

Patients suffering from traumatic head injury, whose Glasgow comma score (GCS) was less than 10 (moderate to severe head trauma), were included in the study. The exclusion criteria were age less than 18 and more than 65 years, history of anti-platelet medications, previous chronic diseases like liver diseases, malignancy, heart diseases, chronic obstructive air diseases, and hypertension, diabetes.

**Table 1:** Comparing the nutritional status indices between groups at different days of admission to intensive care unit

**Nutritional index**	**Serum Adiponectin level (mg/L)**		**P-value**
	**<15 (n = 25)**	**≥15 (n = 39)**	
**Body mass index (BMI)**			
Day 1	28.17 ± 4.05	27.90 ± 3.16	0.77
Day 6	28.02 ± 4.18	26.96 ± 2.9	0.24
Day 13	28.01 ± 4.15	26.46 ± 2.89	0.08
**Fat body mass (FBM)**			
Day 1	25.0 ± 5.09	28.15 ± 6.35	0.05
Day 6	24.6 ± 5.9	26.74 ± 5.9	0.16
Day 13	24.56 ± 5.8	25.46 ± 5.8	0.55
**Lean body mass (LBM)**			
Day 1	50.64 ± 7.03	46.73 ±6.69	0.02
Day 6	50.19 ± 6.95	45.17 ±6.58	0.005
Day 13	49.71 ± 6.52	44.51 ±6.36	0.002
**Abductor pollicis muscle (APM)**			
Day 1	21.79 ± 4.56	20.96 ± 3.41	0.40
Day 6	21.80 ± 4.56	19.76 ± 2.85	0.03
Day 13	21.82 ± 4.35	19.33 ± 3.04	0.009
**Mid upper arm circumference (MUAC)**			
Day 1	27.02 ± 4.1	27.5 ± 2.4	0.56
Day 6	27.51 ± 3.4	26.57 ± 2.67	0.22
Day 13	27.71 ± 3.39	25.68 ± 2.53	0.008
**NUTRIC score (high risk for malnutrition)**			
Day 1	7 (28.0)	5 (12.8)	0.12
Day 6	2 (8.0)	31 (79.5)	<0.001
Day 13	1 (4.0)	32 (82.1)	<0.001
**SGA score (severely malnourished)**			
Day 1	0 (0.0)	0 (0.0)	0.13
Day 6	0 (0.0)	6 (15.4)	<0.001
Day 13	0 (20.0)	28 (71.8)	<0.001

Data are presented as mean ± standard deviation or number (%)

**Table 2:** Comparing the severity of disease between groups at different days of admission to intensive care unit

**Severity of disease**	**Serum Adiponectin level (mg/L)**		**P-value**
	**<15 (n = 25)**	**≥15 (n = 39)**	
**APACHE II score **			
Day 1	12 (4)	19 (6)	0.006
Day 6	12 (4)	12 (5)	<0.001
Day 13	14 (4)	23 (6)	<0.001
**SOFA score **			
Day 1	8 (4)	22 (7)	<0.001
Day 6	8 (4)	14 (5)	<0.001
Day 13	10 (3)	16 (4)	<0.001

Data are presented as median (IQR). SOFA: Sequential Organ Failure Assessment (SOFA) score; APACHE II: Acute Physiology and Chronic Health Evaluation.

***Assessments***

In order to monitor the patients’ malnutrition status, the NUTRIC score (nutrition assessment in critically ill) and SGA score (Subjective global assessment) were calculated within 24 hours from their admission, and then on the 6^th^ and 13^th^ days of their stay in ICU. The patients’ nutritional status consisted of assessing their actual body weight through bed scale (Balas digital body scale, Tehran, Iran), and body composition analysis (fat body mass (FBM) and lean body mass (LBM)) via bioelectrical impedance analysis (BIA) (Body stat’s, London) which was measured within 24 hours after their admission, and on the 6^th^ and 13^th^ days of their stay in the ICU. Moreover, a flexible measuring tape was used to measure their mid-upper arm circumference (MUAC) and a Lange caliper (UK) was utilized in both hands of the patients in order to measure the thickness of adductor pollicis muscle (APM) within 24 hours after their admission, and on the 6^th^ and 13^th^ days of their stay in the ICU.

In order to measure the severity of the disease and incidence of organ dysfunction, Sequential Organ Failure Assessment (SOFA) score and Acute Physiology and Chronic Health Evaluation (APACHE II) scoring systems were employed within 24 hours after admission, and on the 6^th^ and 13^th^ days of stay in the ICU.

***Serum adiponectin measurement***

Within 24 hours after admission in ICU, 5 cc venous blood samples were collected from all patients, in the morning, in order to assess the concentration of serum adiponectin. Afterwards, the blood samples were put on ice and centrifuged at 3000 ×g, and the obtained sedimented plasma was frozen at -70 °C. After the blood samples were collected, immune-assay measurements were carried out in accordance with the manufacturer’s instruction by employing multiplexing technology as fully-automated evidence and semi-automated benchtop analyzer evidence investigator provided by Shanghai Korain Biotech Co. Ltd. which specializes in antibodies, lab supplies of ELISA kit, and protein for life science research. For this purpose, Intra-Assay was considered as CV<8% and Inter-Assay as CV<10%.

***Data gathering***

A check list consisting of patients’ age; gender; GCS; malnutrition status based on NUTRIC and SGA; nutritional status based on BMI, LMM, APM, FBM, and MUAC; serum adiponectin level; and severity of disease (APACHE II and SOFA) was collected for all patients on days 1, 6, and 13 after admission to ICU. A PhD student was responsible for data gathering. 

***Statistical analysis:***

Data were analyzed using ‘‘SPSS version 22 for Windows (SPSS Inc. Chicago, IL, USA)’’. Mean ± standard deviation or number (%) was used to describe the characteristics of the participants. Kolmogrov-Smirnov test was used to examine the normal distribution of the continuous variables. Chi-square test was used for comparison of categorical variables (NUTRIC score, and SGA score) between patients with serum adiponectin level of <15mg/L and ≥15 mg/L. Independent samples T test was applied to compare the continuous data (BMI, fat body mass, lean body mass, APM, and MAUAC) between the two groups at 24hours after admission, day 6 and day 13 of staying in the ICU. Mann–Whitney U test was applied to compare the non-parametric variables (APACHE, and SOFA score) between the two groups. A two-sided p value of 0.05 was considered statistically significant. 

## Results

***Baseline characteristics of studied patients***

Sixty-four patients with the mean age of 35.97 ± 11.5 (20 – 64) years were studied (59.4% male; 57% traffic accidents). Most of the patients (54 (84%)) had less/low risk of malnutrition on admission. The average BMI of the studied participants on admission was 28.01 ± 3.51 and their MAUC, APM, fatty body mass, and lean body mass were 27.3 ± 3.2, 21.2 ± 3.8, 26.9 ± 6.35 and 48.2 ± 7.04, respectively. The patients’ average APACHE and SOFA scores were 16.17 ± 5.07 and 11 ± 3.57, respectively. The mean serum adiponectin of patients was 21.9 ±11.8 (4.2 – 48. 4) mg/L (60.9% of cases ≥ 15 mg/L). 

***Adiponectin and nutritional status***

Table 1 compares the nutritional status indices between head trauma patients with serum adiponectin <15mg/L and ≥15 mg/L, at day1, 6, and 13 of admission to ICU. The nutritional status of head trauma patients with serum adiponectin ≥15 mg/L significantly deteriorated from day 1 to 13 based on different nutritional status indices. BMI (p = 0.08), LBM (p = 0.002), APM (p = 0.009), and MUAC (p = 0.008) had significant decreasing trends from day 1 to day 13 in patients with serum adiponectin level ≥ 15 mg/L. In addition, the number of high risk patients for developing malnutrition based on NUTRIC score (p < 0.001) and the number of severely malnourished cases based on SGA score (p < 0.001) significantly increased from day 1 to 13 in this group. It should be noted that the severity of disease based on APACHE (p < 0.001) and SOFA (p < 0.001) scores increased in the mentioned cases during the follow up period (table 2).

## Discussion

Based on the findings of the present study, serum adiponectin level ≥ 15 is associated with significant deterioration of nutritional status, increase in the risk of malnutrition, and worsening of the clinical outcome in patients with moderate to severe head trauma in ICU.

Evidence on experimental studies demonstrated a consistent influence of adiponectin on inducing either weight loss or weight gain (8). The presence of a clear cause and effect relationship between reducing the weight and increase in the concentration of serum adiponectin has not been confirmed in humans. 

In pre-dialysis chronic kidney disease patients, high serum adiponectin was independently related to protein energy wasting and decrease in muscle mass (9). High level of adiponectin was associated with worse nutritional status of patients with renal disease and suggested to contribute to the pathogenesis of undernutrition in such patients (10). High adiponectin level has also been suggested to have a role in the pathogenesis of cachexia in heart failure (7). 

In metabolic disorders (anorexia and obesity) the level of adiponectin is inversely associated with BMI and fat mass (11). However, the increased concentration of adiponectin in anorexia nervosa was found to be less than what we reported in our head trauma patients. Adiponectin concentration was found to be decreased in obese individuals who tried to reduce their weight by dieting or gastric surgery (12). However, the concentration of plasma adiponectin had not decreased in non-obese healthy people who tried to lose weight (13).

Plasma adiponectin level was also found to be related to worsening of the patients’ outcome. High serum adiponectin level is associated with increased 1-week mortality in those with intra-cerebral haemorrhagic disease (14). In acute respiratory illnesses the high level of serum adiponectin was found to correlate with the severity of the illness and increased mortality of the patients (15). In patients with coronary artery disease, high level of adiponectin was found to be related with an increase in adverse cardiovascular outcomes (16). 

In traumatic brain injury, high level of adiponectin was found to be independently associated with the worse clinical outcome and severity of the disease in traumatic brain injury (5). In agreement with other studies, we found that high level of adiponectin significantly correlated with severity of the disease and the clinical outcome. APACHE score, which reflects the severity of trauma was found to be high in patients who had higher levels of adiponectin. In addition, SOFA score, which reflects organ dysfunction, was also found to be significantly high in patients with adiponectin ≥ 15 mg/L. 

 Some inflammatory mediators have been implicated to be linked to severity and clinical outcomes of traumatic brain injury. These inflammatory mediated cytokines are found to increase after traumatic head injury (17, 18). The anti-inflammatory effect of adiponectin is exerted by blocking the pro-atherogenic process in endothelial cells and releasing AMP- activated kinase, increasing sensitivity to insulin and reducing the risk of glucose tolerance (19). 

Adiponectin has been shown to exert anti-inflammatory and cerebral protective effects. Clear evidence has shown the inverse relationship between several inflammatory markers and plasma adiponectin. Adiponectin has been shown to modulate signalling pathways in certain types of cells (20). Impairment and dysfunction of the organ is a result of inflammatory reaction process during ischemia reperfusion injury (21, 22). Nitric oxide and AMPK are two molecules of cellular signal transduction, which are considered to be involved in adiponectin’s cardio-protecting activities (23). Therefore, the main part of adiponectin’s beneficial effect on cerebrovascular injury is its anti-inflammatory effect, ameliorating and reducing the production of APO, IL-8, IL1b, and TNF (24). 

It seems that serum adiponectin level could be considered as a good screening tool for detection of patients at risk for developing malnutrition and poor outcome following head trauma. Therefore, finding high risk patients and ameliorating their nutritional status may be a useful strategy to improve the outcome of patients with moderate to severe trauma who are admitted to ICU. We recommend a large study to show/investigate the cause and effect relationship between adiponectin and nutritional status in head trauma patients. 

The inclusion of both male and female patients with 13 days follow- up, the application of various methods to assess the nutritional status, and anthropometric measurement are among the strengths of this study. In addition, the actual weight of the patients was assessed using bed scale for the first time in Iraq.

## Limitations

One of the limitations of the current study was measuring serum adiponectin once, on admission. Measuring serum adiponectin at different stages and times could provide better understanding of the behaviour of this adipocytokine. Another limitation was the small sample size of the current study, investigating the association of serum adiponectin with nutritional status in a large sample size might have more accurate results. 

## Conclusion:

Based on the findings of the present study, serum adiponectin level ≥ 15 is associated with significant deterioration in nutritional status, increase in the risk of malnutrition, and worsening of the clinical outcome in patients with moderate to severe head trauma in ICU.

## Availability of data and material:

All data are available on request**.**
